# An Optical Overview of Poly[***μ***_2_-L-alanine-***μ***_3_-nitrato-sodium(I)] Crystals

**DOI:** 10.1100/2012/620189

**Published:** 2012-04-01

**Authors:** E. Gallegos-Loya, E. Orrantia-Borunda, A. Duarte-Moller

**Affiliations:** ^1^Universidad Tecnológica de la Zona Metropolitana de Guadalajara Tlajomulco de Zúñiga, Jaisco, Mexico; ^2^Centro de Investigación en Materiales Avanzados, S. C., Miguel de Cervantes 120, Complejo Industrial Chihuahua, 31109 Chihuahua, CHIH, Mexico

## Abstract

Single crystals of the semiorganic materials, L-alanine sodium nitrate (LASN) and D-alanine sodium nitrate (DASN), were grown from an aqueous solution by slow-evaporation technique. X-ray diffraction (XRD) studies were carried for the doped grown crystals. The absorption of these grown crystals was analyzed using UV-Vis-NIR studies, and it was found that these crystals possess minimum absorption from 200 to 1100 nm. An infrared (FTIR) spectrum of single crystal has been measured in the 4000–400 cm^−1^ range. The assignment of the observed vibrational modes to corresponding symmetry type has been performed. A thermogravimetric study was carried out to determine the thermal properties of the grown crystal. The efficiency of second harmonic generation was obtained by a variant of the Kurtz-Perry method.

## 1. Introduction

Some organic compounds exhibit large NLO response, in many cases, orders of magnitude largest than widely known inorganic materials. They also offer the flexibility of molecular design and the promise of virtually an unlimited number of crystalline structures [1–3]. A number of such crystals, especially from the amino acids family, have recently been reported [4–8]. Some crystals of the amino acids with simple inorganic salts appear to be promising materials for optical second harmonic generation (SHG) [9].

The amino acids display specific features of interest, such as (i) molecular chirality, which secures acentric crystallographic structures; (ii) absence of strongly conjugated bonds, leading to wide transparency ranges in the visible and UV spectral regions; (iii) zwitterionic nature of the molecule, which favours crystal hardness [9]. Further to that, amino acids can be used (iv) as chiral auxiliaries for nitroaromatics and other donor-acceptor molecules with large hyperpolarizabilities and (v) as a basis for synthesizing organic-inorganic compounds [3].

A series of studies on semiorganic amino acid compounds such as L-arginine phosphate (LAP), L-arginine hydrobromide (L-AHBr), L-histidine tetrafluoroborate (L-HFB) [3], L-arginine hydrochloride (L-AHCl) [5], L-alanine acetate (L-AA) [6], and glycine sodium nitrate (GSN) [7, 10] as potential NLO crystals have been reported. L-alanine is an amino acid, and it forms a number of complexes on reaction with inorganic acid and salts to produce an outstanding material for NLO applications.

The compound poly[*μ*_2_-L-alanine-*μ*_3_-nitrato-sodium(I)], [Na (NO_3_) (C_3_H_7_NO_2_)]_n_ [10], was obtained as the product of an attempted reaction of sodium nitrate and the amino acid L-alanine in aqueous solution. In the present investigation, single crystals of poly[*μ*_2_-L-alanine-*μ*_3_-nitrato-sodium(I)] and D-alanine sodium nitrate were grown and characterized by single crystal X-ray diffraction, Fourier transform infrared (FTIR) and high resolution Raman, HR-Raman, spectroscopic studies, thermogravimetric analysis (TGA/DSC), UV-Vis-NIR spectral analysis, and second harmonic generation (SHG).

These compounds have medicinal features that cover a variety of important biological activities, such as the inhibition of specific enzymes or antiviral and antitumor activity [11, 12]. When used in combination with *β*-lactam antibiotics, polyoxotungstates enhance the antibiotic effectiveness against otherwise resistant strains of bacteria [13]. The heptamolybdate, in particular the [NH_3_Pri]6[Mo7O24]*·*3H_2_O, had shown a potent *in vivo *antitumor activity, which has been explained by repeated redox cycles of [Mo7O24]6 in the tumor cells [14].

The compounds, L-alanine sodium nitrate and D-alanine sodium nitrate, were obtained as the product by reaction of sodium nitrate and the amino acids L-alanine and D-alanine in aqueous solution. In the present investigation, single crystals were grown and characterized by X-ray diffraction powder, Fourier transform infrared (FTIR) spectroscopic studies, thermogravimetric analysis (TGA/DTA), UV-Vis-NIR spectral analysis, and the efficiency of second harmonic generation (SHG).

## 2. Experimental Details

The crystals obtained during the development of this work were grown by slow evaporation technique at room temperature through an aqueous solution. The reactive commercially available L-alanine and D-alanine C_3_H_7_NO_2_ were used with stoichiometry Sigma-Aldrich lab with 98% purity and molecular weight 89.09 g/mol and the sodium nitrate NaNO_3_ stoichiometry Sigma-Aldrich lab with 99.9% purity and molecular weight 84.99 g/mol. The samples were prepared 1 : 1 molar ratio in distilled water and constant agitation for 35 min and a temperature of 60°C. The evaporation time for the L-alanine sodium nitrate solution at room temperature was 45 days and 60 days for the D-alanine solution.

The Phillips Expert powder X-ray diffractometer with Cu K*α* radiation (*λ* = 1.5428 Å) was used for the powder X-ray diffraction pattern. The sample was scanned in the 2*θ* values ranging from 10 to 60 degrees at the rate of 0.05°/min.

In order to analyze the presence of functional groups, FTIR spectrum was recorded in the range of 400 cm^−1^ to 4000 cm^−1^ using a MAGNO IR 750 series II NICOLET spectrometer. The samples were added to a matrix of KBr to perform this procedure.

The UV-Vis spectra give limited information about the structure of the molecule because the absorption of UV and visible light involves promotions of the electrons in the π* and σ* orbitals from the ground sate to higher energy states. The transition spectra is very important for any NLO material because it can be of practical use only if it has a wide transparency window. NLO materials have a practical use only if they have a wide transparency state. To find this absorbance window, a Lambda 10 Perkin Elmer UV-Vis spectrometer was used. The scanning was done in the range of 200 to 1100 nm the same way as with the FTIR.

TGA/DSC was done in a TA Instruments STD 2960 Simultaneous TGA/DSC. The samples were heated from room temperature to more than 1000°C at rate of 10°C/min.

In order to find the SHG, the crystals were grown according to the Kurtz and Perry technique [9] into powder (about 70 *μ*m) and densely packed between two transparent microscope glass slides [15, 16]. Once the samples were placed into the glass slides, a Nd:YAG Quanta ray INDI series laser of 1064 nm generating an 8 ns pulse and operated at 6 mJ/pulse and at rate of 10 Hz is pumped at the proper angle and distance in order to see SHG on green color (532 nm) that corresponds to the expected emitted light and it is the half wavelength of the incoming light.

## 3. Results and Discussion

### 3.1. X-Ray Diffraction Analysis

**Figure 1:**
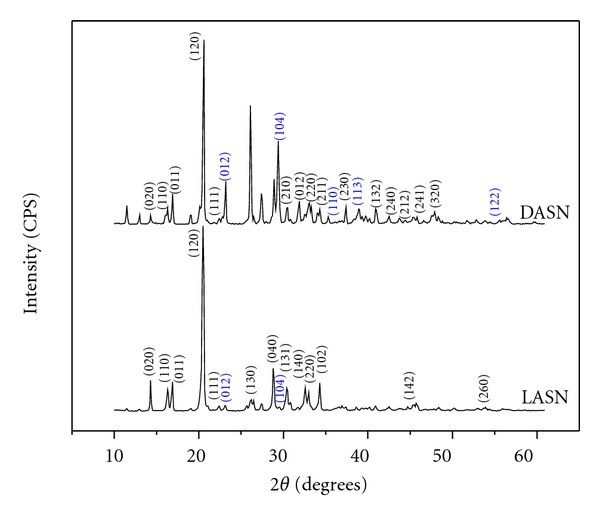
Powder X-ray diffractogram of L-alanine sodium nitrate (LASN) and D-alanine sodium nitrate (DASN).

The resultant peaks in the powder X-ray pattern displayed in Figure 1 shows an intense peak at 20.54°, which coincides with the plane (120) and the reflections of the planes (020), (110), (040), (140), (111), (220), (102), (131), (142), and (260), corresponding to the principal planes of the L-alanine present in the L-alanine sodium nitrate, where the planes (012) and (104) were identified with nitrate. The peaks appearing in the spectrum that have not been identified can be attributed to the formation of the L-alanine sodium nitrate compound. In both the L-alanine and D-alanine cases, the presence of an intense peak at 20.59° which coincides with the plane (120) and reflections from planes (020), (110), (011), (111), (210), (012), (220), (211), (230), and (241), corresponding to the principal planes of the D-alanine present in the D-alanine sodium nitrate, was identified. The planes (012), (104), (110), (113), and (122) were identified with nitrate. The peaks appearing in the spectrum that have not been identified can be attributed to the formation of the compound D-alanine sodium nitrate.

### 3.2. Thermal Analysis

**Figure 2:**
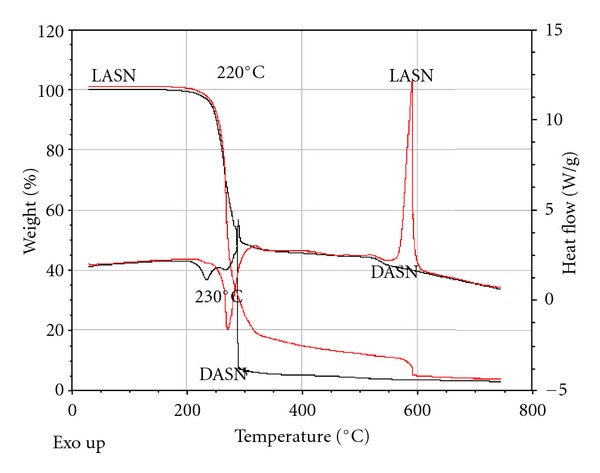
TGA and DSC curves of LASN and DASN.

Figure 2 shows the TGA pattern of the LASN and DASN showing good stability below 220°C with a rapid dropping beyond that temperature [8, 17]. Also, Figure 2 shows the DSC pattern of LASN where an exothermic transition appears at about 230°C. Mean while, the pure presents another endothermic transition at 400°C [9, 17]. Within this temperature range, the possible NLO applications become promising due to the use of laser powers, for LASN and DASN performing below 230°C.

### 3.3. FT-IR Study

In order to obtain the presence of functional groups, FTIR spectrum was recorded in the range of 400 cm^−1^ to 4000 cm^−1^ by using a MAGNO IR 750 series II Nicolet spectrometer.

**Figure 3:**
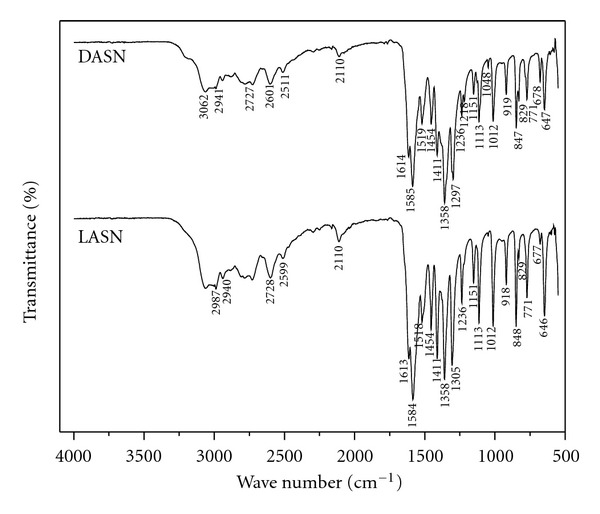
FTIR spectrum of LASN and DASN.

The samples of L-Alanine sodium nitrate (LASN) and D-alanine sodium nitrate (DASN) were added to a matrix of KBr to perform this procedure as shown in Figure 3. The presence of the carboxyl acid group around 3000 cm^−1^ can be observed due to the alanine presence. The main internal vibrations of alanine are observed on the functional groups (NH_3_^+^, CH_2_, COO^−^) which is in agreement with the data reported before [9], symmetric and asymmetric bending vibrations were observed on the CH_3_ groups for LASN and DASN at 2987 and 1454 cm^−1^. The peak at 2599 and 2601 cm^−1^ is a symmetrical stretching CH to LASN and DASN, respectively. The 1151, 1218, and 1236 cm^−1^ frequencies are attributed to the rocking deformation of the NH_3_^+^ group [5]. Furthermore, the peak 1048 cm^−1^ is a symmetrical stretching of CCN group.

Other low frequency bands are typical for N–H*⋯*O hydrogen bonds arising from the overtones around the 2727 cm^−1^. The rest of the functional groups COO^−^, CN, and NO_3_ between 500 and 1500 cm^−1^ also agree with the reported data.

Usually, the presence of nitrates in the lattice can be identified by their characteristic signature in the ranges 1660–1625, 1300–1255, 870–833, and 763–690 cm^−1^ [18]. Parent compound traces were identified in the synthesized compound. The presence of the NO_3_ group in the LASN and DASN can be identified by the peaks at 1358, 1113, 849, and 771 cm^−1^. The symmetric and asymmetric NH_3_^+^ stretching vibrations appear at frequencies 2941 and 2110 cm^−1^, respectively. The absorption peaks at 1613, 1584, and 1518 cm^−1^ for LASN and 1614, 1585, and 1519 cm^−1^ for DASN confirm the presence of NH_3_ bending. The presence of nitro-groups in the spectrum confirms the LASN and DASN compounds. Other important functional groups are detailed in Table 1.

### 3.4. Raman Spectroscopy

**Figure 4:**
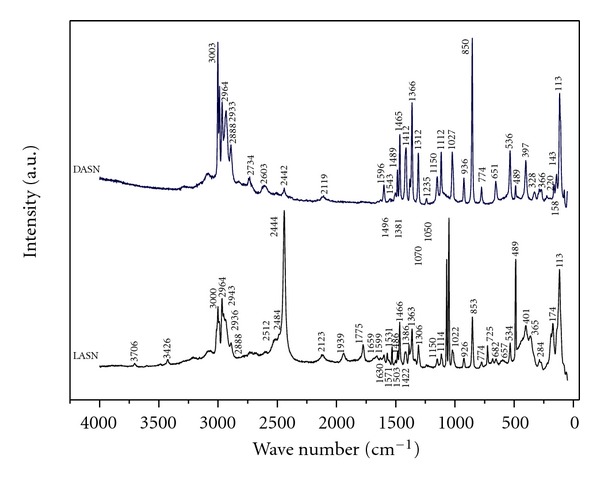
Raman spectrum of LASN and DASN.

The Raman spectra was carried out at room temperature in frequency range 400–4000 cm^−1^ by using a LabRAM H-R Raman microscope HORIBA system. The laser Raman spectrum, showing the presence of more intense peak around 850 cm^−1^, is due to COO^−^ stretching mode of vibrations (see Figure 4). The peaks at 1113 and 1112 cm^−1^ are assigned to NO_3_ stretching.

The C–H and N–H bending vibrations are observed at 1306 cm^−1^ as a sharp peak. The asymmetric CH_3_ bending at 1422 cm^−1^ and O–H bending is around 950 cm^−1^ [19]. The peak at 1411 cm^−1^ is assigned to the symmetric stretching C–COO carboxyl group.

In Raman spectra of alanine, the symmetric and asymmetric deformation vibrations of the NH_3_^+^ groups appear in the region between 1680 and 1470 cm^−1^ [20]; in the spectrum L-alanine and D-alanine, we found in 1531 y 1939, 1659, 1630, 1599, 1596 cm^−1^ frequency. The position of NH_3_^+^ asymmetric stretching frequency indicates the formation of intra- and intermolecular strong N–H*⋯*O hydrogen bonding of the NH_3_^+^ group, with the oxygen of both, the carbonyl group and inorganic nitrates [10, 21]. The study of symmetry stretching and stretching vibration of CH_2_ group is observed in 2948 and 2964, 2888 cm^−1^. The band around 1235, 1150, 1151, and 926 cm^−1^ is also indicative of the NH_3_ rocking modes. The peak at 1366 cm^−1^ is a deformation of CH_2_ group, at 1312 cm^−1^ is attributed to the CH_2_ wagging. The intensity varies upon the source used for analyzing the sample. Other important functional groups are detailed in Table 1.

### 3.5. UV-Vis Study

**Figure 5:**
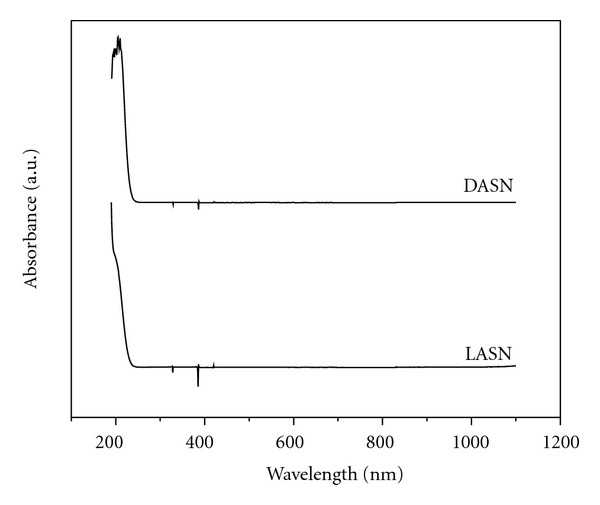
UV-Vis window of the LASN and DASN.

Figure 5 presents the absorbance zone above 250 nm (ultra-violet wavelength) where a wide band completely transparent in all the visible range is observed (infrared wavelengths) [9, 19, 22]. This means that this material presents a good nonabsorbance band in the visible range for expected applications. A little protuberance around the 300 nm is observed [7]. This little peak is still outside the visible zone (UV zone), and it could present some absorbance if the crystal was to be excited with 600 nm (red color) trying to obtain a second harmonic of 300 nm (UV color). Other noticeable characteristic in the absorption spectrum is a wide transparency window within the range of 400–1100 nm which is desirable for NLO crystals because the absorptions in an NLO material near the fundamental or second harmonic signals will lead to the loss of the conversion of SHG. Due to this property, LASN and DASN have potential uses for SHG using an Nd:YAG laser (1064 nm) to emit a second harmonic signal within the green region (532 nm) of the electromagnetic spectra.

### 3.6. SHG Signal Detection

**Figure 6:**
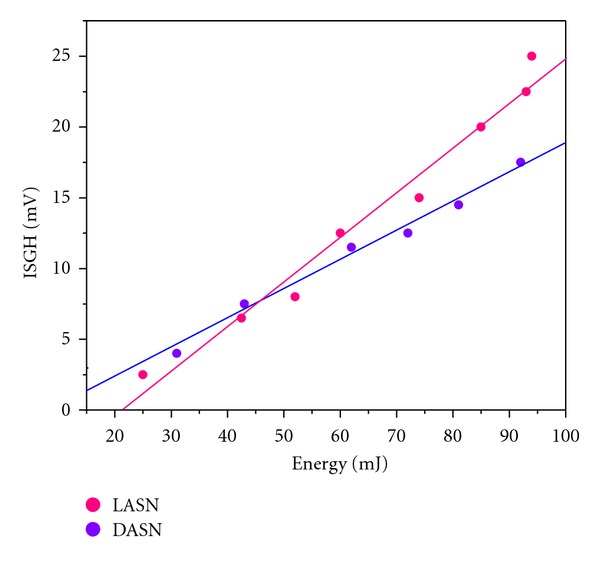
Quadratic fit for the SHG intensity as function of the beam energy.

Figure 6 shows the data collected from the detector where the SHG signal is plotted versus the beam energy. This kind of experiments has been used in order to measure the damage threshold. In this case, the SHG intensity tends to increase when the beam energy is also increased. This experiment shows the good quality of these crystals for the second harmonic generation, but there is a better efficiency in the LASN sample.

## 4. Conclusions

A new nonlinear optical semiorganic crystal, LASN, and DASN were grown by the slow evaporation technique from aqueous solution. Functional groups of good quality crystals of LASN and DASN have been detected by FTIR and Raman spectroscopies.

Also based on UV-Vis spectra observations, an absorption zone below the 250 nm (ultra-violet wavelengths) can be seen recovering a good transmittance values across all the visible range until near IR frequencies and beyond. This situation shows these crystals can be used for applications involving the band of visible light. The transparency of the crystal in the visible and infrared regions shown in transmission spectra confirms the NLO property of this.

Other characterization was the thermal response. The TGA/DSC results showed a degrading temperature about 230°C which promises to have good applications at high temperatures, revealing that the crystal is thermally stable until that temperature.

The SHG test is the first one performed in this kind of material, and it was observed that the SHG intensity tends to be directly proportional to the beam energy and follows a linear tendency with a positive slope which promises to be a good nonlinear optical material.

Finally, we conclude that we succeeded in obtaining good quality crystals of LASN and DASN, and, for the first time, the second harmonic generation was detected in this material which indicates that these crystals are new materials with nonlinear optical properties with potential applications.

## Figures and Tables

**Table 1:** FT-IR and Raman functional group assignments of the grown LASN and DASN.

RAMAN (cm^−1^)		FTIR (cm^−1^)		Assignments
LASN	DASN	LASN	DASN	
3706				Overtone
3426				Overtone
			3062	Symmetric CH_3_ stretching
3000	3003			Asymmetric CH_3_ stretching
		2987		Symmetric CH_3_ stretching
2964	2964			CH_2_ stretching and asymmetric CH_3_ stretching
2948				Symmetric CH_2_ stretching
		2941	2941	Symmetric NH_3_ stretching
2936	2933			Asymmetric CH_2_ stretching
2888	2888			CH_2_ stretching
	2734			Overtone
		2728	2727	N–H*⋯*O and O–H*⋯*O stretching
	2603		2601	Symmetric CH stretching
		2599		Symmetric CH stretching
2512			2511	Overtone
2484				Overtone
2444	2442			Overtone
		2251		CH_3_ stretching
2123	2119			Overtone
		2110	2110	Asymmetric NH_3_ stretching
1939				Asymmetric NH_3_ deformation
1775				Asymmetric COO stretching
1659				Asymmetric NH_3_ deformation
1630				Asymmetric NH_3_ deformation
		1613	1614	NH_3_ bending
1599	1596			Asymmetric NH_3_ deformation
		1584	1585	NH_3_ bending
1571				Asymmetric COO^−^ stretching
	1543			Overtone
1531				Symmetric NH_3_ deformation
		1518	1519	NH_3_ bending
1503				CH_3_ deformation
	1496			Overtone
1486	1489			Asymmetric COO^−^ deformation
1466	1465			C_*β*_H_2_ scissors mode
		1454	1454	Asymmetric CH_3_ bending
1422				CH_3_ bending
	1412	1411	1411	Symmetric C–COO^−^ stretching
1386				CH_3_ puckering
1363	1366			Wagging CH_2_ deformation
		1358	1358	NO_3_ stretching
	1312			CH_2_ wagging
1306		1306		C–H and N–H bending
			1297	Flexed position CH_2_
	1235	1236	1236	NH_3_^+^ rocking
		1218	1218	NH_3_^+^ rocking
1151	1150	1151	1151	NH_3_^+^ rocking and symmetric COO^−^ stretching
1113	1112	1113	1113	NO_3_ stretching
1070				Overtone
1050		1048	1048	Symmetric CCN stretching
1022	1027			CH_3_ rocking
		1012	1012	Overtone of torsional oscillation NH_3_^+^
926				NH_3_ rocking
	936			CH_2_ rocking
		918	919	Overtone of torsional oscillation NH_3_^+^
853	850			N–C stretching
		849	847	NO_3_ stretching
		829	829	C–C stretching
774	774			OH deformation
		771	771	NO_3_ stretching
725				COO wagging
		677	678	NO_3_^−^ in plane deformation
	651	646	647	COO^−^ in plane deformation
		578	578	Overtone
